# Impact of Social Determinants of Health on Post-operative Outcomes Following Robotic Radical Prostatectomy

**DOI:** 10.1007/s11912-025-01728-5

**Published:** 2025-11-01

**Authors:** Faris Najdawi, Samuel Lassiter, Alina Gandrabur, Ryan W. Dobbs, Mohammed Shahait

**Affiliations:** 1 Division of Urology, Cook County Health and Hospitals System, Chicago, IL United States; 2 Department of Urology, University of California at Irvine, 19200 Jamboree Road Suite 5100, Irvine, CA United States

**Keywords:** Robotic-assisted, Prostatectomy, Social determinants of health

## Abstract

**Purpose:**

Social determinants of health are increasingly recognized as key contributors to disparities in healthcare access and outcomes. With robotic-assisted radical prostatectomy now widely adopted as the preferred surgical approach for localized prostate cancer, this systematic review evaluates how individual social determinants of health influence access to robotic surgery and postoperative outcomes.

**Materials and Methods:**

This review adhered to PRISMA guidelines and was registered with PROSPERO (CRD420256270179). A comprehensive search of PubMed and EBSCO identified studies examining social determinants of health in patients undergoing robotic prostatectomy. Extracted data included patient demographics, social determinants of health variables, and perioperative outcomes. Risk of bias was assessed using the Cochrane Risk of Bias Tool and the Newcastle-Ottawa Scale.

**Results:**

Eighteen studies met inclusion criteria. Commonly assessed variable included socioeconomic status, race/ethnicity, insurance, education, occupation, and geographic location. Lower socioeconomic status was linked to decreased robotic prostatectomy access, treatment at low-volume or non-robotic centers, and worse outcomes. Racial and ethnic disparities were consistent; non-White patients were less likely to receive definitive therapy and more likely to undergo surgery by low-volume providers. Rural patients experienced reduced access to robotic surgery and lower rates of pelvic lymph node dissection. Lower education levels were associated with delayed continence and reduced return-to-work capacity.

**Conclusions:**

Social determinants of health significantly impact access to robotic prostatectomy and postoperative outcomes. Urologists and policymakers should integrate awareness of these factors into patient counseling and institutional planning. Future research should explore mechanisms underlying these disparities to inform equity-driven strategies in prostate cancer care.

## Introduction

Social determinants of health (SDOH) are non-medical environmental factors that influence healthcare access, health outcomes, and quality of life [1]. These include socioeconomic conditions, where individuals are born or live, race/ethnicity, occupation, and geographic context. According to the Centers for Disease Control and World Health Organization, SDOH also encompass broader structural forces like economic and social policies [2]. The PROGRESS-Plus framework was developed to define SDOH [3]. It defines SDOH as Place of residence, Race/ethnicity, Occupation, Gender, Religion, Education, Socioeconomic status (SES), Social capital, plus age, disability, and sexual orientation [4]. A large body of evidence has shown the powerful influence of SDOH in perpetuating population health inequities [5].

Prostate cancer is the fourth most diagnosed cancer globally and a leading cause of cancer deaths in men. In the United States, most men present with localized disease [6, 7]. Robotic-assisted radical prostatectomy (RARP) is now the standard surgical treatment [8]. Despite uniform guidelines and wide availability to robotic technology, outcome disparities persist, often driven by SDOH. Investigating how SDOH influence RARP outcomes is essential to reduce inequities and guide future care.

## Methods

This systematic review followed PRISMA guidelines and was registered with PROSPERO (CRD420256270179) [9].

### Evidence Acquisition

Comprehensive literature searches were conducted on PubMed and EBSCO databases. Articles included discussed at least one SDOH and its effect on RARP outcomes. Given the paucity of data, absolute exclusion criteria were not applied to ensure a comprehensive list of available literature. Search terms included (“prostate cancer” OR “robotic assisted radical prostatectomy” OR “RARP” OR “RALP”) AND (“social determinants of health” OR “place of residence” OR “insurance” OR “socioeconomic” OR “occupation” OR “employment status” OR “race” OR “ethnicity” OR “religion” OR “education” OR “social capital” OR “social determinants”). Two authors independently screened titles and abstracts. Disagreements were resolved by a senior reviewer. Outcomes of interest included complications, 30-day readmission, length of stay (LOS), quality of life (QOL), and satisfaction. Variables analyzed included race/ethnicity, SES, community type, employment, insurance, and education. Included studies are summarized in Table 1.

**Table 1:** Summary of included studies

Author, year	Country	Study Design	Level of Evidence	Sample Population	Sample Size	Follow-up	SDOH Reported	Outcomes Reported
Schroeck, 2008	USA	Cross-Sectional Study	II	Men undergoing RRP	400	1 year	Race, Ethnicity, Employment Status, Education level	Complication Rate, Pt satisfaction of those who received RARP vs. ORRP
Nilsson, 2011	Sweden	Prospective Cohort Study	II	Men undergoing RRP	1288	2.2 years	Employment status, Education level, Age, BMI, Comorbidities	Urinary Continence
Parsons, 2014	USA	Retrospective Cohort	III	Men undergoing RARP	401,325	5 years	Race/Ethnicity, SES, Insurance status	Minimally invasive prostatectomy vs. Open prostatectomy, Complication Rate
Kim, 2015	USA	Cross-Sectional Study	II	Men undergoing RRP	20,411	3 years	Place of Residence, Race, SES, Insurance Status	Treatment with RARP
Potretzke, 2016	USA	Retrospective Cohort	III	Men undergoing RARP	274	30 days	Age, Race, Comorbidities, BMI	LOS, 30 Day Complication Rate, 30 Day Readmission Rate
Gerhard, 2017	USA	Retrospective Cohort	III	Men with High-risk prostate Cancer	60,300	6 months	Place of Residence (rural vs. Urban), Race, Education level, SES, Insurance status,	Offered to receive definitive treatment, Use of Technologically advanced treatment (RARP and IMRT)
Mechow, 2018	Germany	Retrospective Cohort	III	Men undergoing RRP	1415	1 year	Employment status, SES, Demands of Job	Time to return to work
Dahl, 2020	Norway	Cross-Sectional Study	II	Men with Prostate Cancer	730	3 years	Age, Employment status, Comorbidities, Educational level	Self-reported workability status post RARP
Xia, 2021	USA	Retrospective Cohort	III	Men who underwent RARP	115,355	3 years	Place of Residence, Race/Ethnicity, SES, Insurance Status	Receipt of BL PLND at time of RARP
Pathak, 2021	USA	Retrospective Cohort	III	Men undergoing RARP	49,238	30 days	Comorbidities (BMI), Race	Complication Rate, 30 Day Readmission, LOS
Noel, 2022	UK	Retrospective Cohort	III	Men undergoing RARP	682	5 years	Race, BMI	Complication Rate, 30-day Readmission, LOS, Oncologic Outcome, Potency, Urinary Continence
Mourao, 2022	Brazil	Retrospective Cohort	III	Obese vs. Non-obese men undergoing RARP	1077	2 years	Comorbidities (BMI)	Post-operative urinary continence, Complication Rate
Hajj, 2022	USA	Retrospective Cohort	III	Men undergoing RARP	31,253	30 days	Comorbidities (BMI, CHF, dialysis, smoking status), Race	LOS
Farzat, 2023	Germany	Retrospective Cohort	III	Obese vs. Non-obese undergoing RARP	500	1 year	Comorbidities (BMI)	Complication Rate, 30-day Readmission rate, Oncological Result
Logan, 2023	USA	Retrospective Cohort	III	Men undergoing RRP	243,292	1 year	Place of Residence, Race, Education Level, SES, Insurance status,	Treatment with RARP vs. Open RRP
Mao, 2024	USA	Cross-Sectional Study	II	Men with prostate cancer	18,926	3 years	Race, Insurance Status	Treatment with RARP vs. ORRP
Mao, 2024	USA	Observational Study	IV	Men undergoing RARP	18,926	30 days	Race, Educational Level, SES, Insurance Status,	Complication Rate, 30-day Readmission rate, LOS, Treatment with RARP vs. ORRP
Mossack, 2024	USA	Retrospective Cohort	III	Men undergoing RARP	100,035	60 days	Insurance status, SDOH in general	60-day Complication Rate

### Quality Assessment

Bias in randomized clinical trials (RCT) was evaluated using the Cochrane Risk of Bias Tool [10]. Non-randomized studies used the Newcastle-Ottawa Scale. Extracted data from individual articles and their risk of bias scores were recorded by the authors in a custom data template for analysis. Data were collected using Excel software (Microsoft Corporation, Redmond WA version 12.2.4).

## Results

**Fig. 1:**
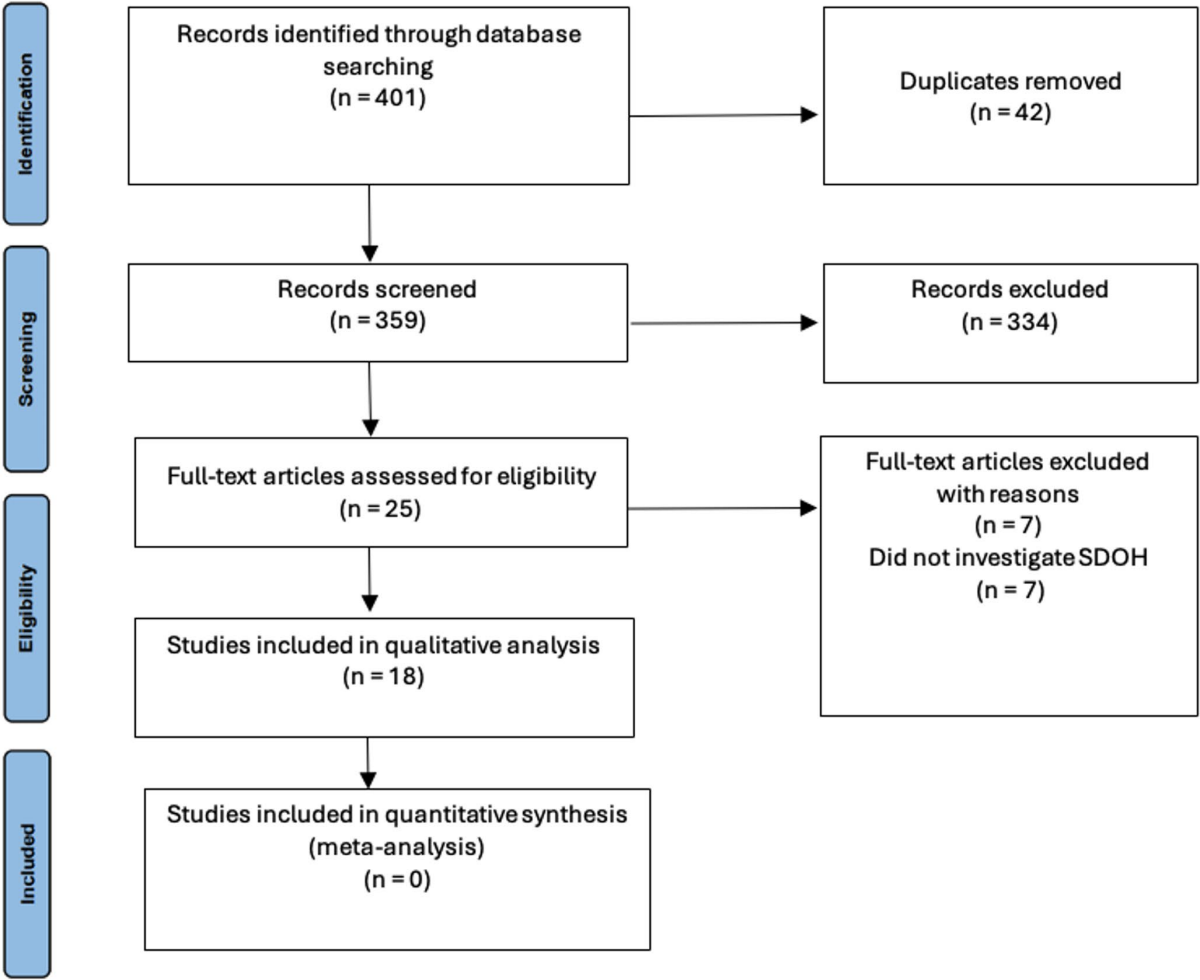
Prisma flow diagram

From 401 articles, 18 studies (2008–2024) met inclusion criteria: 12 retrospective cohorts, 4 cross-sectional, 1 observational, and 1 prospective. No available studies were RCT. Due to heterogeneity of reported variables, meta-analysis was unable to be performed. Our search strategy is outlined in Fig. 1.

### Socioeconomic status

Five primary studies (retrospective cohort [*n* = 4], cross-sectional [*n* = 1]) assessed the relationship between SES and outcomes related to RARP [11–15]. We found SES was measured and reported in multiple ways across each study analyzed. Measures varied considerably including: individual-level annual household income, area-level indicators such as median zip code income, and median county income quartiles. We have highlighted these distinctions in the Results to enhance clarity when interpreting available data and specified the SES definition used in each included study in Table 2.

**Table 2:** Summary of varying measurements in reporting SES

Author, year	Measurement	Definition of low/high SES
Xia, 2021	Area-based	Median household income for each patients’ area of residence (zip code): Lowest quartile = <$38,000; Highest quartile = >$63,000
von Mechow, 2018	Patient reported	Monthly income: *≤*2000 €; 2000–2999 €; 3000–3999 €; *≥*4000 €
Parsons, 2014	Area-based	Median household income (zip code): <$45,000 vs. *≥*$45,000
Kim, 2015	Area-based	Median household income (zip code). Quartile 1–4, without defined dollar amount
Gerhard, 2017	Area-based	Median income based on census tract: Lowest quartile = < $38,000; Highest quartile = >$63,0000

### Choice of Treatment

A higher SES was associated with higher likelihood to undergo RARP versus open radical prostatectomy (ORP) when compared to their lower SES counterparts [13]. Separately, the association between SES and undergoing RARP was shown with data analyzed from the Healthcare Cost and Utilization Project, the largest publicly available all-payer inpatient care database in the United States containing 5 to 8 million community hospital stays annually. It found that patients with an annual income greater than $45,000 were more likely to undergo RARP compared to those with an income less than $45,000 (OR 1.28, p-value < 0.001) [13].

### Access to Surgery

Lower SES patients, defined as those residing in areas characterized by the lowest quartile of income, are more likely to be treated at a facility that does not have the capability to perform RARP when compared to their higher SES counterparts, defined as those living in areas with the highest quartile of income [14]. Additionally, patients with lower SES are less likely to be offered definitive therapy such as radical prostatectomy (RP) or radiation therapy for treatment of their high risk prostate cancer [15]. In this study, lower SES was defined as the lowest quartile of median annual household income (<$38,000) compared to the highest quartile (>$63,000).

### Perioperative Outcomes

When defining SES based on monthly income, patients with a lower SES are associated with a longer duration of time taken off from work post-RP^12^. Though this analysis combined RARP and ORP data, the authors separately reported surgical approach (ORP versus RARP) did not affect return to work time on Cox proportional hazard analysis. Furthermore, this association incrementally increases in strength as monthly income increases. Patients in a lower SES group were also reported to undergo lower rates of pelvic dissection (PLND) at the time of RARP, when defining SES based on median household income for each patients’ zip code [11].

## Occupational Status

Four primary studies (cross-sectional [*n* = 2], retrospective cohort [*n* = 1], prospective cohort [*n* = 1]) evaluated the association between occupational status and outcomes related to RARP [12, 16–18]. Patients who are working full-time prior to undergoing RARP are more likely to report their post-operative workability as ‘high’ compared to those who are retired or working part-time [18]. Moreover, self-employed patients took a shorter duration of leave compared to patients who were employed under a company. Additionally, patients who described their job as “heavy workload” took a significantly longer period of leave following RARP compared to patients who identify their workload as light or moderate [12]. A study by Schroeck et al. attempted to identify factors associated with satisfaction and regret following radical prostatectomy, including both ORP and RARP. On multivariate analysis, they did not identify an association between employment status and frequency of regret or dissatisfaction following radical prostatectomy, neither in ORP or RARP. Regarding patient-reported outcomes, those who were employed were more likely to achieve continence when compared to retired and disabled patients at 2 years post-operatively [16, 17] No available studies evaluated occupational status to type of treatment or access to surgery.

### Place of Residence

Four primary studies (retrospective cohort [*n* = 3], cross-sectional [*n* = 1]) evaluated the association between place of residence (urban versus rural) and outcomes related to RARP [8, 11, 14, 15]. Patients treated at “low technology” facilities were more likely to be offered non-definitive management [15]. Specifically, patients living in rural counties were less likely to receive RARP as definitive therapy for their prostate cancer when compared to individuals in metropolitan counties [8]. Furthermore, patients living in non-metropolitan areas were more likely to be treated at a non-RARP performing hospital and, therefore, less likely to be offered minimally invasive therapy via RARP compared to their metropolitan counterparts [14]. Finally, patients in rural areas are less likely to undergo PLND at time of RARP when compared to their urban counterparts, and both urban and rural patients are far less likely to undergo BPLND when compared to patients in metropolitan areas [11].

### Race and Ethnicity

Ten primary studies (retrospective cohort [*n* = 7], cross-section [*n* = 2], observational [*n* = 1]) evaluated the association between race/ethnicity and outcomes related to RARP [8, 11, 13, 15, 16, 19–22]. Non-white men with high-risk prostate cancer are less likely to receive definitive therapy for their disease when compared to white men [15]. When evaluating type of definitive surgical treatment, White and Asian patients are more likely to receive RARP, rather than ORP, when compared to Hispanic and non-Hispanic Black patients [8, 13]. Furthermore, Black and Hispanic patients who did undergo RARP were more likely to have had their surgery performed by a low-volume RARP surgeon compared to their white counterparts [23]. Hispanic patients are also less likely to undergo BPLND at the time of RARP, but this was not seen for non-Hispanic black patient [11]. With regards to immediate postoperative course, non-White patients are more likely to have a prolonged LOS when compared to their white counterparts [19]. Additionally, Hispanic and black patients have higher rates of post-operative complications and 30-day readmissions which could, in part, be explained by a lower rate of RARP compared to ORP [20]. Of note, two studies analyzing outcomes of high-volume RARP surgeons reported conflicting results. They reported no difference in LOS, complications, potency and urinary continence between different ethnic groups [21, 22]. With regards to patient satisfaction, Black patients were more likely to regret their treatment decision [16].

### Insurance Status

Five primary studies (retrospective cohort [*n* = 3], cross-sectional [*n* = 1], observational [*n* = 1]) evaluated the relationship between insurance status and patients who underwent RARP [8, 14, 15, 20, 23]. Patients with Medicaid or uninsured were less likely to undergo routine screening for prostate cancer. The same population was also more likely to opt for non-definitive management (NDM) of high-risk prostate cancer (HRCaP) compared to patients with Medicare or private insurance (14.5% vs. 27.7%; OR 1.97). Moreover, non-white HRCaP patients with Medicaid or uninsured managed at low-tech facilities were seven times more likely to receive NDM, compared to well-insured White men managed at high-tech facilities (OR 7.18; 95% CI 5.37–9.61) [15]. Having Medicare or private insurance in addition to being treated at a high-tech facility diminished this disparity, reducing the odds to 1.5 times as likely to undergo NDM, regardless of the patient’s race [15]. Regardless of prostate cancer risk group, patients with Medicaid or uninsured were associated with decreased odds of undergoing RARP when compared to Medicare or privately insured patients [8, 14, 20, 23].

### Education

Four studies analyzed the relationship between postoperative outcomes and patient education (prospective [*n* = 1], cross-sectional [*n* = 3] [8, 16–18]. Nilsson et al. reported patients with a low educational level had an increased age-adjusted relative risk (RR) of developing long-term urinary incontinence after radical prostatectomy when compared to patients with high education levels (RR 2.5; 95% CI, 1.7–3.9) [17]. In their study, low education was defined as *≤* 12 years of schooling, whereas high education was defined as *≥* 13 years of schooling, and urinary incontinence was defined as use of *≥* 2 pads daily. Their analysis included both RALP and ORP but did not stratify results.

Dahl et al. sought to identify variables associated with moderate/low work ability after RARP. They found patients with low education were at significantly increased risk of low/moderate work ability postoperatively when compared to high education patients, both on univariate (OR 2.09, *p* < 0.001) and multivariate (OR 1.84, *p* = 0.001) analyses. Their study population was on average three years post-RALP [18].

Interestingly, Schroeck et al. reported there was no statistically significant difference in patient rate of satisfaction and rate of regret after radical prostatectomy between high and low education patients. These results did not stratify ORP versus RARP patients. Though, after adjusting for stage, Gleason Score, and PSA in multivariate logistic regression analysis, lower income was ultimately found to be independently associated with treatment satisfaction following radical prostatectomy (*p* < 0.001) [16].

Disparities in access to and clinical outcomes of RARP were reported by analyzing patient data in the National Cancer Database (NCDB). A total of 354,752 patients who underwent radical prostatectomy in the United States between 2010 and 2017 were included, with 297,676 (83.9%) of them receiving RARP, while 57,076 (16.1%) underwent ORP. Though patient-specific income was not reported, those living in areas characterized by the lowest quartile of education were less likely to receive RARP compared to those living in areas with the highest quartile of education (81.4% vs. 85.5%; *p* < 0.001) [8].

## Discussion

We identified several SDOH influencing the likelihood of patients undergoing RARP for prostate cancer, including SES, place of residence, race and ethnicity, education, and insurance status. These factors were also associated with worse postoperative outcomes, prolonged time to return to work, and higher rates of patient regret. This systematic review highlights the substantial impact of SDOH on both access to RARP and perioperative outcomes.

Lower SES was consistently linked to decreased access to RARP. Patients with lower SES were less likely to be offered definitive treatment and, when they were, more likely to undergo ORP instead of RARP [14, 15]. Contributing factors may include limited access to screening, delayed diagnoses, and increased prevalence of comorbidities, which make these patients less optimal surgical candidates [24, 25]. Moreover, they are often treated at hospitals lacking robotic capabilities [14]. These barriers are critical, as RARP has been associated with improved outcomes, including reduced transfusion rates, shorter hospital stays, fewer readmissions, and quicker return to work [8, 20, 23]. Innovations have even enabled same-day discharge for select RARP patients, a benefit not available with ORP [26].

Place of residence also influences access to robotic surgery. Patients in rural or non-metropolitan areas are less likely to undergo RARP and more likely to receive care at low-volume facilities, which are associated with poorer outcomes [23]. Additionally, these patients are less likely to receive PLND, a key component of high-risk prostate cancer management [14].

Race and ethnicity also significantly impact access to RARP. White and Asian patients are more likely to undergo RARP than Hispanic and Black patients. This disparity is partially due to minority patients receiving care at hospitals that do not offer robotic surgery [14]. Furthermore, Black patients undergoing RARP are more often treated by low-volume surgeons, which may contribute to longer hospital stays and lower satisfaction rates.

Occupational status affects recovery and postoperative function. Patients employed at the time of surgery tend to return to work sooner and have higher continence rates than retired or unemployed individuals. Those with physically demanding jobs often require longer recovery. These patterns suggest that employment status not only reflects functional reserve but also influences motivation and adherence to postoperative care, such as pelvic floor exercises.

Occupational status in the U.S. frequently intersects with education and insurance, further complicating the relationship between SDOH and outcomes. Patients with Medicaid or no insurance are less likely to receive routine screening and more likely to experience treatment delays than those with private insurance [27]. Even after adjusting for race and hospital type, privately insured patients are more likely to undergo definitive treatment. Community-level education also plays a role – residing in an area with low average education levels correlates with reduced access to RARP. Patients with lower education levels who do undergo RARP report worse outcomes, particularly lower postoperative continence rates. These differences may be partly explained by challenges in understanding and executing perioperative care instructions, as prior studies have shown that patient-centered perioperative education improves outcomes such as continence, length of stay, and postoperative sexual function [28, 29]. Interestingly, satisfaction rates did not differ significantly by education level.

Understanding the influence of SDOH on healthcare access and surgical outcomes is essential for improving both patient care and health policy. There is ongoing debate in health policy circles regarding whether outcome measures, including the public reporting and reimbursement systems built upon them, should account for SES or other SDOH [30]. Advocates argue failing to adjust for these factors unfairly penalizes hospitals serving a disproportionate number of low-SES patients. Opponents caution that such adjustments may obscure true disparities in care quality [31].

Further research is needed, particularly prospective studies, to more accurately measure the impact of SDOH on RARP outcomes and identify modifiable barriers. In clinical practice, urologists should recognize and account for these factors during preoperative counseling and planning. Tailored interventions, including enhanced education and access to high-volume surgical centers, may help reduce disparities and improve outcomes for all patients undergoing RARP.

### Limitations

Due to the rapidly evolving landscape of robotic surgery, some findings may be outdated. Studies outside the U.S. limit generalizability. No low- or middle-income country data was included, limiting applicability. Social capital (a PROGRESS-Plus factor) wasn’t assessed due to limited literature. Variations in measuring SES across studies limits direct comparison and synthesis of findings. Heterogeneity of data prevented pooled analysis.

## Conclusion

SES, race/ethnicity, insurance, education, occupation, and area of residence significantly influence RARP access and outcomes, contributing to worse post-operative outcomes and decreased patient-reported satisfaction. Urologists and policymakers must recognize these disparities and implement strategies to reduce their impact. Future research should aim to further define and address these critical SDOH in prostate cancer surgery.

## Key References

- Logan CD, Mahenthiran AK, Siddiqui MR, et al. Disparities in access to robotic technology and perioperative outcomes among patients treated with radical prostatectomy. Journal of surgical oncology. 2023;128(2):375-384.
  - One of the largest reviews conducted of patients who underwent RARP, identifying several key factors inhibiting access to robotic surgery.
- Mossack SM, Franco A, Roadman DF, et al. Social determinants of health and surgical outcomes of minimally invasive radical prostatectomy: a national population-based study. Prostate Cancer and Prostatic Diseases. 2024:1-6.
  - This large-scale review of an insurance claims database provides valuable data on the impact of SDOH post-RARP complications.

## Data Availability

No datasets were generated or analysed during the current study.
